# Jod-Basedow Phenomenon Precipitating Diabetic Ketoacidosis: A Case Report

**DOI:** 10.7759/cureus.83544

**Published:** 2025-05-05

**Authors:** Abin Thomas, Jerin Varghese, Sreekrishnan Trikkur, Gireesh Kumar

**Affiliations:** 1 Department of Emergency Medicine, Amrita Institute of Medical Sciences and Research Center, Kochi, IND

**Keywords:** diabetic keto acidosis, hyperthyroidism, iodine, iodine-induced hyperthyroidism, jod-basedow effect, thyroiditis

## Abstract

Jod-Basedow phenomenon (JBP) is an alternative term for iodine-induced thyrotoxicosis, which is a rare hyperthyroid illness that is brought on by excessive iodine exposure. Patients with underlying thyroid conditions, autoimmune diseases, or chronic renal disease are populations frequently affected by the JBP. The potential for this disorder to circumvent normal thyroid autoregulation and result in potentially fatal consequences makes it especially worrying.

Here, we describe the case of a 50-year-old woman who had a known case of dyslipidemia, cardiomyopathy, and type 2 diabetes and presented to the emergency department with complaints of nausea and stomach pain. Diabetic ketoacidosis (DKA) was suspected based on venous blood gas reports that showed significant anion gap metabolic acidosis. Imaging revealed characteristics indicative of thyroiditis, and additional testing verified hyperthyroidism. Interestingly, the patient had only had iodinated contrast medium coronary angiography one day before presentation to the emergency department. This case highlights the underdiagnosed yet severe risk of iodine-induced thyroid dysfunction, particularly in patients with silent thyroid disease, which can precipitate acute conditions such as DKA.

## Introduction

Jod-Basedow phenomenon (JBP) is a rare thyrotoxic syndrome produced by excessive extrinsic iodine exposure, commonly known as iodine-induced hyperthyroidism (IIH) [1]. However, JBP is not limited only to cases of excessive iodine exposure. Among iodine-deficient individuals, JBP was present; however, this was contributed to by an acute surge in iodine levels [2,3]. Now, a shift in paradigm is observed among the individuals affected with JBP, as more cases of JBP are among individuals exposed to Iodinated Contrast Medium (ICM). Individuals who are exposed to ICM, especially for radiological tests and treatments, are reported to have developed JBP [2-4].

The underlying mechanism for the development of JBP is contributed by the excess of iodine. According to the Institute of Medicine, the United Nations Children’s Fund, the World Health Organization, and the International Council for the Control of Iodine Deficiency Disorders, the accepted amount of iodine per individual is 150 micrograms per day [3,5]. However, according to Apsan and Antal, contrast media contain 13,500 μg of free iodine and 15-60 g of bound iodine [3]. Hence, following procedures involving exposure to ICM, the patient’s body is exposed to high levels of iodine, which can contribute to JBP. However, to compensate for this iodine overload, our body has an autoregulatory mechanism called the Wolff-Chaikoff effect [4].

The Wolff-Chaikoff effect is a transient hypothyroid state generated to counteract the iodine overload. However, a certain percentage of individuals, particularly those with pre-existing thyroid disorders, can bypass this auto-regulatory action, resulting in the development of JBP [2]. Hypothyroidism brought on by the Wolff-Chaikoff effect is typically temporary and often returns to normal levels within 48 hours [3]. However, some patients may experience an increase in thyroid hormone production, thereby causing thyrotoxicosis and, consequently, JBP - especially among individuals with pre-existing thyroid disease - thus necessitating close observation [2,4].

Following the development of JBP, among patients with diabetes mellitus, a hypermetabolic state is marked by elevated lipolysis, glucose production, and high consumption of energy [6]. A relative insulinopenic state brought on by greater insulin clearance and glucose absorption in hyperthyroidism can lead to diabetic ketoacidosis (DKA) [7]. Here, we report a rare case of exposure to ICM leading to the development of a life-threatening scenario of DKA due to JBP.

## Case presentation

A 50-year-old woman with a known history of type 2 diabetes mellitus, dyslipidemia, and cardiomyopathy arrived at the emergency department with complaints of vomiting and abdominal pain for the past day. She had a past medical history of consuming oral metformin and atorvastatin. On physical examination, her abdomen was soft and non-tender. She was initially managed with antiemetics, analgesics, and intravenous (IV) fluids. Venous blood gas was done, which is depicted in Table 1, and it showed features of a high anion gap metabolic acidosis. Following the identification of high anion gap metabolic acidosis, DKA was suspected, and serum ketones were sent, which came out to be positive. Hence, a provisional diagnosis of DKA was made. The patient was managed with IV fluids, and an evaluation for a possible precipitating factor was done. There was no history of consuming any new drugs.

**Table 1:** Venous blood gas analysis pH: acid-base balance of blood; PCO_2_: partial pressure of carbon dioxide; HCO_3_: bicarbonate level

Venous blood gas (VBG)	Lab values	Normal values
pH	7.009	7.35-7.45
PCO_2_	12.2 mmHg	35-45 mmHg
HCO_3_	2.9 mmol/L	22-28 mmol/L
Lactate	1.8 mmol/L	0.5-2.2 mmol/L

**Figure 1:**
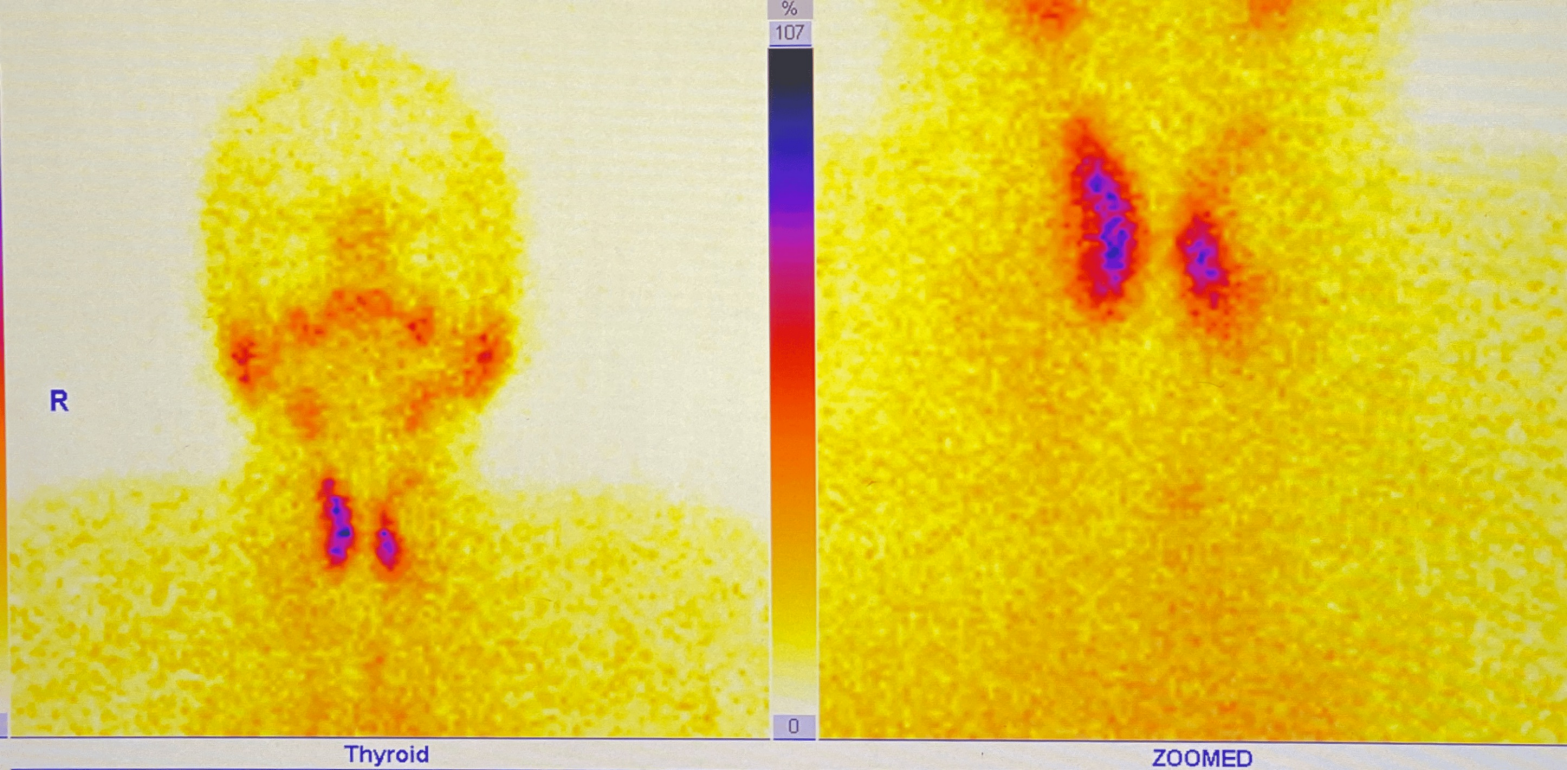
Technetium scintigraphy showing mild uptake

Interestingly, WBC counts were 8440 K/uL, and CRP was 12 mg/L, within standard ranges. After an abdominal computed tomography (CT) scan, cholelithiasis was discovered without any signs of cholecystitis. On further assessment, the patient’s serum thyroid levels were found to be high and were suggestive of thyroiditis. Laboratory findings of the thyroid function test (TFT) have been depicted in Table 2. While trying to insert an external jugular vein for IV fluid administration, a palpable mass was identified on the patient's neck. Hence, an ultrasound of the neck was done, and it showed features of thyroiditis and multinodular goiter (MNG). Using the American College of Radiology Thyroid Imaging Reporting and Data System (ACR TIRADS), three nodules were identified. Upon suspecting thyroiditis, subsequently, anti-thyroid peroxidase (anti-TPO) and anti-thyroglobulin antibody (anti-Tg) were performed, which have been depicted in Table 2. Technetium thyroid scintigraphy was done for additional assessment, and it showed findings suggestive of thyroiditis (Figure 1). Her routine thyroid evaluation was done one year back, and thyroid-stimulating hormone levels were normal (1.74 mU/mL).

**Table 2:** Thyroid function test fT4: free thyroxine 4; fT3: free thyroxine 3; anti-TPO: anti-thyroid peroxidase; anti-Tg: anti-thyroglobulin antibody

Test	Result	Reference range
TSH	0.02 mU/mL	0.4-4.0 mU/mL
fT4	27.6 pg/mL	0.8-1.8 pg/mL
fT3	7.9 pg/mL	2.3-4.2 pg/mL
anti-TPO	253 U/mL	0-35 U/mL
anti-Tg	361 U/mL	0-40 U/mL

Given the history of coronary angiography with iodine dye one day before admission to our emergency department, which was done in view of stress cardiomyopathy, along with altered TFT values and thyroid scintigraphy showing features suggestive of thyroiditis. This is a rare, underreported phenomenon called JBP, and hence, a diagnosis of DKA caused by the JBP was finalized. The patient was started on anti-thyroid medication and managed conservatively for DKA. The patient was clinically stable and was discharged after eight days of in-hospital admission.

## Discussion

The thyroid gland, when exposed to excessive amounts of iodine, produces the acute Wolff-Chaikoff effect. This acute effect is partially explained by the synthesis of molecules like iodolipids, iodolactones, or iodoaldehydes that inhibit the activity of thyroid peroxidase (TPO), which is necessary to produce thyroid hormone [8]. The escape from this effect is called JBP. JBP is a rare thyrotoxic condition caused by increased exogenous iodine exposure, also known as IIH [9]. In patients at risk, excess iodine intake could result in either acute or chronic hyperthyroidism [10,11].

IIH is frequently associated with silent thyroid illness; however, elderly people may not exhibit classic hyperthyroidism symptoms [12,13]. Excess thyroid hormone increases a metabolic process called lipolysis, which involves the breakdown of body fat into serum-free fatty acids and the secretion of insulin and glucagon. The sum of these effects causes glucose metabolism to worsen, which causes glucose intolerance and diabetes.

The combined effect of insufficient insulin and high counterregulatory hormones causes lipolysis, the release of free fatty acids into the circulation from adipose tissue, and uncontrolled hepatic fatty acid oxidation in the liver, which creates ketone bodies and ketonemia. It is due to increased gluconeogenesis, faster glycogenolysis, and poorer peripheral tissue glucose uptake [14].

DKA is a life-threatening condition and requires prompt management to prevent severe decompensation [15]. In patients with underlying, untreated hyperthyroidism, DKA can be precipitated by factors like metabolic derangement, drugs, stress, burns, or infections [16]. In a study by Breuel et al., 39 German patients who had initially been euthyroid were shown to have substantially elevated serum T3 levels and lower TSH levels after being exposed to ICM [17]. Conn et al. identified a predisposition toward hyperthyroidism following non-ionic contrast imaging among 73 patients [18]. Our case describes the occurrence of contrast-induced thyroiditis post-coronary angiography, thyroid dysfunction in the absence of previous dysfunction, and the presence of well-functioning thyroid glands.

A case report by Shah et al. explains the thyrotoxicosis-induced DKA in a patient on insulin who has type 2 diabetes and no prior thyroid history, similar to our case report [8]. In a study by Özkan et al., 56 days after iodine exposure, about 5.8% of the 101 patients who had coronary angiography developed subclinical hyperthyroidism [19]. Other evidence includes a case report by Mushtaq et al., which followed eight iodinated CT scans over 10 months in a 76-year-old man who had previously used amiodarone and developed hyperthyroidism and sudden onset of atrial fibrillation [20]. Our case also suggests the development of DKA post-thyroiditis.

## Conclusions

IIH, a rare manifestation of JBP, can occur following coronary angiography. Although iodine contrast-induced thyroiditis is frequently underdiagnosed, it can have serious side effects, such as DKA. This example highlights the importance of early diagnosis and treatment, especially for patients who have just had contrast imaging treatments when they present with acute metabolic disturbances. Despite the thyroid's prior normal function, the iodine contrast exposure caused significant thyroid hormone dysregulation. These problems must be recognized and treated immediately to prevent potentially deadly outcomes.
